# An Amine‐Functionalized Iron(III) Metal–Organic Framework as Efficient Visible‐Light Photocatalyst for Cr(VI) Reduction

**DOI:** 10.1002/advs.201500006

**Published:** 2015-02-09

**Authors:** Li Shi, Tao Wang, Huabin Zhang, Kun Chang, Xianguang Meng, Huimin Liu, Jinhua Ye

**Affiliations:** ^1^ Graduate School of Chemical Sciences and EngineeringHokkaido UniversitySapporoJapan; ^2^ International Center for Materials Nanoarchitectonics (MANA), and Environmental Remediation Materials UnitNational Institute for Materials Science (NIMS)1‐1 NamikiTsukubaIbarakiJapan; ^3^ TU‐NIMS Joint Research CenterSchool of Materials Science and EngineeringTianjin University92 Weijin RoadNankai DistrictTianjin300072P.R. China; ^4^ Collaborative Innovation Center of Chemical Science and Engineering (Tianjin)Tianjin300072P.R. China

**Keywords:** Cr(VI) reduction, electron transfer, metal–organic frameworks, photocatalysts

## Abstract

The photocatalytic reduction of Cr(VI) is investigated over iron(III)‐based metal–organic frameworks (MOFs) structured as MIL‐88B. It is found that MIL‐88B (Fe) MOFs, containing Fe_3_‐μ_3_‐oxo clusters, can be used as photocatalyst for the reduction of Cr(VI) under visible light irradiation, which is due to the direct excitation of Fe_3_‐μ_3_‐oxo clusters. The amine‐functionalized MIL‐88B (Fe) MOFs (denoted as NH_2_–MIL‐88B (Fe)) shows much higher efficiency for the photocatalytic Cr(VI) reduction under visible‐light irradiation compared with MIL‐88B (Fe). It is revealed that in addition to the direct excitation of Fe_3_‐μ_3_‐oxo clusters, the amine functionality in NH_2_–MIL‐88B (Fe) can also be excited and then transferred an electron to Fe_3_‐μ_3_‐oxo clusters, which is responsible for the enhanced photocatalytic activity for Cr(VI) reduction. The enhanced photocatalytic activity for Cr(VI) reduction is also achieved for other two amine‐functionalized iron(III)‐based MOFs (NH_2_–MIL‐53 (Fe) and NH_2_–MIL‐101 (Fe)).

## 1 Introduction

This is an open access article under the terms of the Creative Commons Attribution License, which permits use, distribution and reproduction in any medium, provided the original work is properly cited.

The increasing toxic heavy metal ions contamination in natural water has become a serious issue faced by all the governments in the world, which stimulates intense research on the advanced technology for water treatment.^1^, ^2^ Hexavalent chromium (Cr(VI)) is a typical heavy metal ion contaminant in the waste water arising from some industrial process, such as leather tanning, paint‐making, or steel manufacturing.^3^, ^4^ Cr(VI) is highly toxic to organisms, carcinogenic in human beings, and is mobile in the nature due to its high solubility in water.^5^ Therefore, Cr(VI) has been listed as one of the priority controlled pollutants and its concentration in drinking water has been strictly regulated by many countries. Various techniques for removing Cr(VI) from waste water have been developed, including chemical precipitation, ion exchange, membrane separation, adsorption, and reduction.^6^, ^7^, ^8^

The reduction of Cr(VI) to Cr(III) is considered as an efficient strategy for the removal of Cr(VI) in the waste water, because Cr(III) is environmentally friendly and vital to plants and human life.^5^ Meanwhile, Cr(III) is mostly immobile, which can be precipitated or sorbed on a variety of substrates in neutral or alkaline solutions.^8^ Reduction of Cr(VI) to Cr(III) by semiconductor photocatalysis is a relatively effective method for the removal of Cr(VI) in the waste water. This photocatalytic method is based on the electron–hole pairs (e^−^–h^+^) generated in the semiconductor materials under illumination by light of energy greater than the semiconductor band gap.^9^ These electrons, which migrate to the semiconductor surface, are capable of reducing Cr(VI) to Cr(III) in the solution. Many researchers have reported the photocatalytic reduction of Cr(VI) over TiO_2_.^10^, ^11^, ^12^, ^13^ However, the application of TiO_2_ is limited by its wide band gap (3–3.2 eV), which can only absorb the UV light occupying no more than 5% in solar light.^14^ In recent years, many efforts have been devoted to searching for other efficient photocatalysts that are active under visible‐light irradiation. The photocatalytic reduction of Cr(VI) over visible‐light active photocatalysts such as CdS, SnS_2_, Ag_2_S, and WO_3_ has been widely reported.^15^, ^16^, ^17^, ^18^ But unfortunately, the activity of these photocatalysts for the reduction of Cr(VI) is not very high and the reduction process usually takes a long time. On the other hand, sulfide materials are normally not stable enough to be used as photocatalysts due to the photocorrosion problem, and may cause secondary pollution because of their high toxicity.^19^ Thereby, to explore novel visible‐light active photocatalysts with stable and high photocatalytic ability for the reduction of Cr(VI) is desperately needed.

Metal–organic frameworks (MOFs) are a class of hybrid porous materials composed of metal–oxo clusters and organic building blocks, which have shown a variety of potential applications.^20^, ^21^, ^22^, ^23^ Especially, the application of MOFs in photocatalysis is emerging as an interesting topic recently.^24^ Compared with the traditional photocatalysts, the superiority of MOFs is based on their desirable topology and high surface area, which is beneficial for fast transport and good accommodation of guest molecules. Moreover, the band gap of MOFs is closely related to the HOMO–LUMO gap, which may be flexibly tuned through rational modification of the inorganic unit or the organic linker during synthetic procedures, thus the efficient light harvesting can be realized.^25^ In this context, Gascon et al. observed that the band gap energy of MOF‐5 can be tuned by changing the organic linker.^26^ A theoretical study from Walsh and co‐workers also revealed that the optical response of a Ti‐based MOF could be successfully tuned through rational functionalization of the linking unit.^27^ These studies demonstrated the high potential of MOFs as photocatalysts. In fact, some MOFs such as titanium‐, zirconium‐, and iron‐based MOFs have already been demonstrated to have photocatalytic activities for dye degradation, water splitting, and CO_2_ reduction.^28^, ^29^, ^30^, ^31^, ^32^, ^33^, ^34^ The proposed photocatalytic mechanism involves an photoinduced electron transfer from the photoexcited organic linker to the metal–oxo clusters within MOFs and the direct excitation of metal–oxo cluster.^34^ Despite the great progress achieved so far, the photocatalytic performance of MOFs have not been fully exploited. In particular, little work has been reported on application of MOFs for the photocatalytic reduction of Cr(VI) with high performance and good stability.

Herein, we reported the photocatalytic reduction of Cr(VI) over an amine‐functionalized iron(III) MOF (NH_2_–MIL‐88B) under visible‐light irradiation. We found that NH_2_–MIL‐88B (Fe) exhibited an excellent reusability and much higher photocatalytic activity for Cr(VI) reduction than that of MIL‐88B (Fe) under visible‐light irradiation. Importantly, the visible‐light activity of NH_2_–MIL‐88B (Fe) is even higher than widely used photocatalyst P25 irradiated under UV–visible light. Furthermore, iron(III)‐based MOFs with mixed 1, 4‐benzenedicarboxylic acid (BDC) and 2‐amino‐benzenedicarboxylic acid (BDC–NH_2_) ligands were prepared, and those MOFs all exhibited MIL‐88B structure. The introduction of amine group in iron(III)‐based MOFs can enhance efficiency for the photocatalytic Cr(VI) reduction, the more the better. A dual excitation pathways mechanism for photocatalytic reduction of Cr(VI) over NH_2_–MIL‐88B(Fe) was proposed: i.e., photoinduced exciting of amine functionality followed by electron transfer from the excited organic linker to the Fe_3_‐μ_3_‐oxo clusters and the direct excitation of Fe_3_‐μ_3_‐oxo clusters. The electron transfer phenomenon was revealed by photoluminescence (PL), electron spin resonance (ESR), and transient photocurrent studies. The effect of amine functionalization in the other two iron(III)‐based MOFs (MIL‐53 (Fe) and MIL‐101 (Fe)) on the visible light photocatalytic activity for the reduction of Cr(VI) was also studied. The same trend was observed, namely, all of the amine‐functionalized MOFs showed enhanced activity for photocatalytic Cr(VI) reduction compared with those of their parent MOFs. This work provides us with better understanding of photocatalytic behavior over MOFs photocatalyst and also demonstrates the promising potential of MOFs as high stable and efficient photocatalysts.

## 2 Results and Discussion

### 2.1 Characterization of MOF Photocatalysts

**Figure 1:**
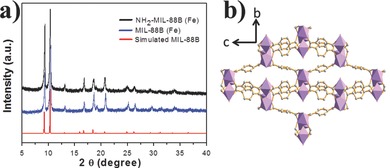
a) XRD patterns of MIL‐88B (Fe), NH_2_–MIL‐88B (Fe), and simulated MIL‐88B; b) MIL‐88B (Fe) structure viewed along *a*‐axis.

**Figure 2:**
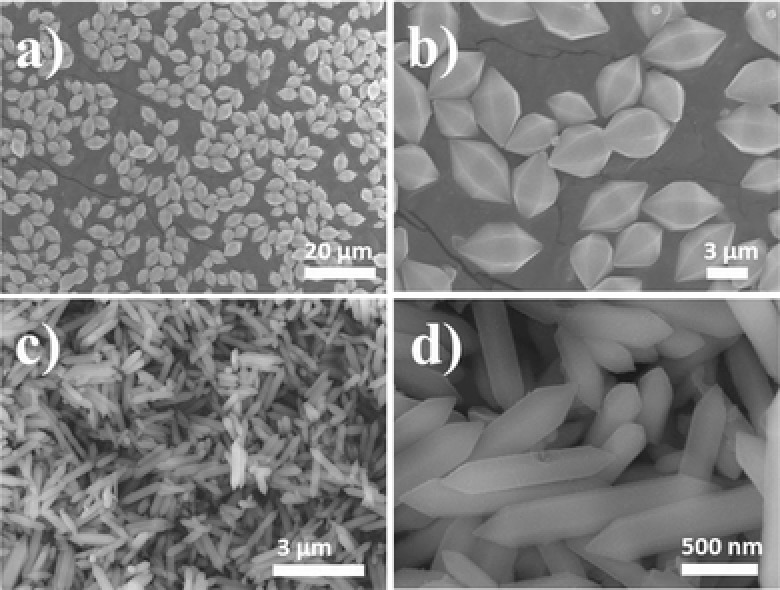
SEM images of a,b) MIL‐88B (Fe) and c,d) NH_2_–MIL‐88B (Fe).

Metal–organic framework MIL‐88B (Fe) and NH_2_–MIL‐88B (Fe) were produced through a rapid microwave‐assisted solvothermal synthesis method. The crystallographic structure of the products was determined by powder X‐ray diffraction (PXRD). As shown in **Figure** **1**a, the well‐defined diffraction peaks revealed the high crystallinity of the products, which are in good agreement with the simulated MIL‐88B structure. The MIL‐88B (Fe) and NH_2_–MIL‐88B (Fe) exhibit the same diffraction pattern, indicating that the crystal phase structure is retained after amine functionalization. The MIL‐88B frameworks are built up from Fe_3_‐μ_3_‐oxo clusters interconnected by oxidation stable terephthalate linkers (Figure 1b). Fe_3_‐μ_3_‐oxo cluster exhibits an octahedral structure with three iron atoms, four oxygen atoms from the bidentate dicarboxylates, one μ_3_O oxygen atom, and one oxygen from terminal ligand (water molecular or halogenide ligand).^35^ The morphology and particle size of these obtained MIL‐88B (Fe) and NH_2_–MIL‐88B (Fe) crystals were observed by scanning electron microscopy (SEM). **Figure** **2**a,b show that MIL‐88B (Fe) crystals have a spindle‐shaped morphology with an average size of 5.5 μm in length and 3.2 μm in diameter. In comparison, the NH_2_–MIL‐88B (Fe) crystals were needle‐shaped with a length of about 1.6 μm and a diameter of 260 nm (Figure 2c,d). In order to elucidate the semiconductor properties of NH_2_–MIL‐88B (Fe) and MIL‐88B (Fe), Mott–Schottky measurements have been conducted. As shown in Figure S1, Supporting Information, the flat‐band potential of MIL‐88B (Fe) is determined to be 0.13 V versus NHE, which is more negative than the Cr(VI)/Cr(III) potential (+1.15 V, pH 3.0).^36^ This indicates that MIL‐88B (Fe) has potential for the reduction of Cr(VI) to Cr(III). The flat‐band potential of NH_2_–MIL‐88B (Fe) is also around 0.13 V versus NHE, which means that the incorporation of amine group in MIL‐88B (Fe) framework has little effect on the conduction band position. This can be explained that the conduction band of iron(III)‐based MIL‐88B MOFs is mainly constructed from Fe 3d and O 2p states and the N 2p state from the amine group participates in the construction of valance band because of the strong electron‐donating characteristics of aromatic amines.^27^

### 2.2 Evaluation of Photocatalytic Activity

**Figure 3:**
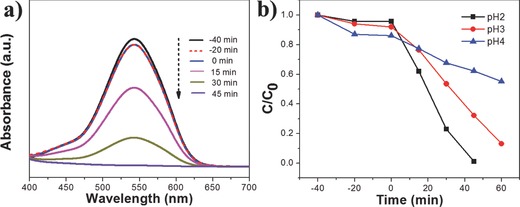
a) Time‐dependent absorption spectral pattern of DPC–Cr(VI) complex solutions after reduction over NH_2_–MIL‐88B (Fe) at pH 2; b) photocatalytic reduction of Cr(VI) over NH_2_–MIL‐88B (Fe) at different pH values. Reaction condition: 20 mg photocatalyst, 40 mL of 8 ppm Cr(VI), reaction temperature is 30 °C, visible light.

The photocatalytic reduction of Cr(VI) was performed over MIL‐88B (Fe) and NH_2_–MIL‐88B (Fe) under visible light irradiation. The photocatalytic reaction was evaluated through monitoring the decolorizating of the UV–vis adsorption spectra of diphenylcarbazide (DPC)–Cr(VI) complex solutions. **Figure** **3**a displays the time‐dependent spectral changes of DPC–Cr(VI) complex solution at various exposure times in the presence of NH_2_–MIL‐88B (Fe) at pH 2. It shows that the reaction solution achieves the adsorption–desorption equilibrium after a presetting of 40 min in the dark. With the increase of irradiation time, the absorption peak at 540 nm ascribed to the DPC–Cr(VI) complex decreases gradually, and it almost disappears after 45 min, which demonstrates the photocatalytic reduction of Cr(VI) over NH_2_–MIL‐88B (Fe). Previous studies reported that the pH value of the solution had a great effect on the reduction rate of aqueous Cr(VI) over photocatalyst.^36^, ^37^ To investigate the influence of pH value on the photocatalytic reduction of Cr(VI), controlled experiments of photocatalytic reduction of Cr(VI) over NH_2_–MIL‐88B (Fe) have been carried out with different pH values. Figure 3b presents the time dependence of the photocatalytic reduction of Cr(VI) catalyzed by NH_2_–MIL‐88B (Fe) at different pH values. The pH value of the K_2_Cr_2_O_7_ solution was adjusted in the range from 2 to 4 by addition of requisite amount of 0.2 m H_2_SO_4_. It shows that the lower pH value results in better photocatalytic performance. This catalyzed effect of the acidified pH values is consistent with the previously reported results that the photocatalytic reduction of Cr(VI) is an acid‐catalyzed behavior.^36^, ^37^, ^38^ At pH 2–4, the predominating species of chromium is Cr_2_O_7_^2−^, and the Cr(VI) reduction reaction could be expressed as follows^36^

(1) $${\mathrm{Cr}}_{2}O_{7}^{2-}+{\mathrm{14H}}^{+}+{\mathrm{6e}}^{-}\to {\mathrm{2Cr}}^{3+}+{\mathrm{7H}}_{2}O$$

(2) $${\mathrm{2H}}_{2}O+{\mathrm{2h}}^{+}\to H_{2}O_{2}+{\mathrm{2H}}^{+}$$

**Figure 4:**
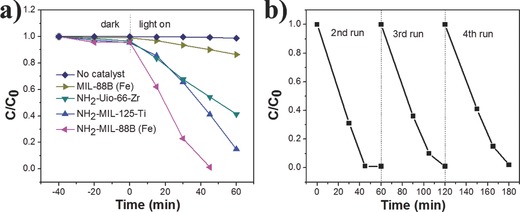
a) Reduction profiles of photocatalytic reduction of Cr(VI) over various photocatalysts; b) recycling test on NH_2_–MIL‐88B (Fe) for photocatalytic reduction of Cr(VI). Reaction condition: 20 mg photocatalyst, 40 mL of 8 ppm Cr(VI), reaction temperature is 30 °C, pH = 2.

The obtained performance of photocatalytic reduction of Cr(VI) over NH_2_–MIL‐88B (Fe) was compared to those of MIL‐88B (Fe), and two typical visible‐light active MOF photocatalysts (NH_2_–Uio‐66–Zr and NH_2_–MIL‐125–Ti). The formations of NH_2_–Uio‐66–Zr and NH_2_–MIL‐125–Ti have been confirmed by their X‐ray diffraction (XRD) patterns (Figure S2, Supporting Information). **Figure** **4**a displays the time dependence of the photocatalytic reduction of Cr(VI) over different types of MOFs photocatalysts. It is obvious that the reduction of Cr(VI) hardly occurred in the absence of photocatalysts and NH_2_–MIL‐88B (Fe) exhibits much higher activity than that of other visible‐light MOFs photocatalysts. The obtained performance of photocatalytic reduction of Cr(VI) over NH_2_–MIL‐88B (Fe) was also compared to those of N‐doped TiO_2_, g‐C_3_N_4_, commercial CdS, and widely used photocatalyst P25 (Figure S3, Supporting Information). It can be seen that the visible light photocatalytic performance of NH_2_–MIL‐88B (Fe) is much higher than N‐doped TiO_2_ and g‐C_3_N_4_. Importantly, NH_2_–MIL‐88B (Fe) also showed even higher visible‐light photocatalytic activity than that of P25, which is conducted under UV–visible light irradiation. This demonstrates that NH_2_–MIL‐88B (Fe) exhibits superior photocatalytic activity for the reduction of Cr(VI).

It is well‐known that the stability and reusability of the photocatalysts are very important from an economic point of view. The used photocatalyst can be recovered by filtration, washing with water and ethanol, and then drying under vacuum. SEM images after photocatalytic reaction at different pH values were measured and shown in Figure S4, Supporting Information. It is obvious that the morphology of NH_2_–MIL‐88B (Fe) can almost retain at pH 2–4. However, the lower pH values (pH 1.7 and 1.5) result in the deterioration of morphology, and NH_2_–MIL‐88B (Fe) can be totally dissolved when the pH value is adjusted to 1. Thus, the photocatalytic performance of NH_2_–MIL‐88B (Fe) was tested under pH 2–4 conditions and the stability of the photocatalysts was further investigated at the reaction condition of pH 2. The XRD pattern of NH_2_–MIL‐88B (Fe) after the photocatalytic reaction reveals almost no deterioration in the crystal structure (Figure S5, Supporting Information), and the Fourier transform infrared (FTIR) spectra of the photocatalyst after the reaction did not show obvious changes (Figure S6, Supporting Information). Inductively coupled plasma analysis of the photocatalyst before and after the reaction revealed that only 0.9% iron ions leak from NH_2_–MIL‐88B (Fe). All of the data indicate the stability of the photocatalyst in this reaction conditions. The X‐ray photoelectron spectroscopy (XPS) results further confirm the stability of the photocatalyst. As shown in Figure S7, Supporting Information, the XPS spectra of samples measured before and after photocatalytic reaction are almost the same. Fe 2p peaks of NH_2_–MIL‐88B (Fe) at binding energy ≈711 and ≈725 eV are assigned to Fe 2p_3/2_ and Fe 2p_1/2_ for iron(III) oxide, which is consistent with the iron(III)‐based MOFs previously reported.^39^ The N 1s XPS spectrum locates at 399.1 eV, and this is the same as BDC–NH_2_ ligand.^40^ Interestingly, in the XPS spectra of NH_2_–MIL‐88B (Fe) after photocatalytic reaction, a weak peak located at 577.1 eV can be observed. This peak corresponds to Cr 2p and resembles the Cr(III) in Cr(OH)_3_.^36^ This further confirms the reduction of Cr(VI) to Cr(III) over NH_2_–MIL‐88B (Fe). Moreover, the weak intensity of Cr 2p XPS signal indicates that the Cr(III) can be removed by simply washing the sample, which is beneficial for reusability of the photocatalyst. We have further studied the reusability of the photocatalyst NH_2_–MIL‐88B (Fe) by collecting and reusing the photocatalyst for four times. As shown in Figure 4b, the photocatalyst NH_2_–MIL‐88B (Fe) can retain its good photocatalytic activity even after four cycles of reaction. Only insignificant decrease in photocatalytic activity is observed, which might be partly caused by loss of photocatalyst during each collection.

### 2.3 Clarification of the Mechanism

Iron(III)‐based MOFs, containing Fe–oxo clusters, have already been demonstrated to have semiconductor properties for the photocatalytic degradation of Rhodamine B, which can be ascribed to the direct excitation of Fe–oxo clusters.^41^, ^42^ In this work, NH_2_–MIL‐88B (Fe) displays much higher visible light photocatalytic performance than MIL‐88B (Fe). Taking into account that MIL‐88B (Fe) and NH_2_–MIL‐88B (Fe) contain the same Fe_3_‐μ_3_‐oxo clusters and reduction potential, the amine group from the linkers contributes to the enhanced photocatalytic performance. Previous studies found that linker modification were beneficial for the UV‐light active MOF photocatalysts based on titania or zirconia clusters, due to the ligand‐to‐metal charge transfer (LMCT) mechanism.^32^, ^33^ The PL has been proved as a powerful technique to study the LMCT mechanism in MOFs.^32^, ^43^ For NH_2_–MIL‐88B (Fe), there also exists the electron transfer from the BDC–NH_2_ organic linker to Fe_3_‐μ_3_‐oxo clusters, and such electron‐transfer process was studied by PL spectra. Actually, as displayed in Figure S8, Supporting Information, when excited at 350 nm, BDC–NH_2_ showed a broad luminescence band centered at 540 nm, which is attributed to its local excitation.^32^ However, NH_2_–MIL‐88B (Fe) did not show any PL signal around 540 nm, thus suggesting the electron transfer from the excited BDC–NH_2_ organic linker to the Fe_3_‐μ_3_‐oxo clusters. To gain further evidence of LMCT process, ESR studies were also carried out. As shown in Figure S9a, Supporting Information, when BDC–NH_2_ was irradiated with visible light, an ESR signal with a *g* value of 2.004 was observed. According to literature, the new signal was produced by the space confined amine group upon irradiation.^32^ In contrast, when NH_2_–MIL‐88B (Fe) was irradiated with visible light, the above signal was not detected. Moreover, as shown in Figure S9b, Supporting Information, NH_2_–MIL‐88B (Fe) shows a typical ESR signal with a *g* value of 1.996 in the dark, which can be ascribed to Fe^3+^.^44^ The ESR signal intensities of Fe^3+^ decreased upon visible light irradiation. Also, when the irradiation was stopped, the intensity of this ESR signal recovered. The decrease of Fe^3+^ ESR signal intensity could be attributed to the trapping of electrons by Fe^3+^ site in Fe_3_‐μ_3_‐oxo clusters.^44^ The disappearance of the ESR signal at *g* value of 2.004 and the decrease of the ESR signal at *g* value of 1.996 suggest the electron transfer from excited amine group to Fe_3_‐μ_3_‐oxo clusters in NH_2_–MIL‐88B (Fe) irradiated with visible light.

**Figure 5:**
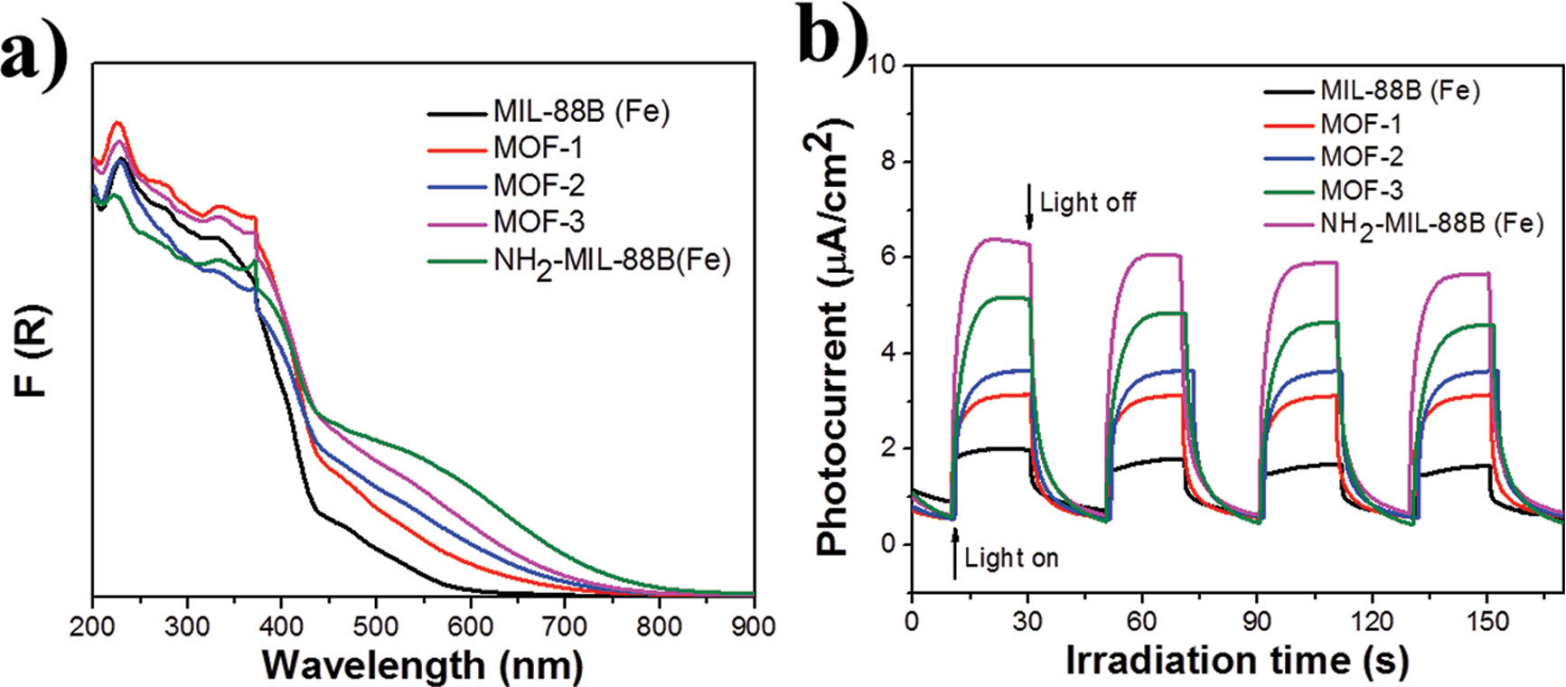
a) The diffuse‐reflectance UV/vis spectrum and b) the transient photocurrent responses of the prepared MIL‐88B (Fe), NH_2_–MIL‐88B (Fe), and mixed MIL‐88B (Fe).

The iron(III)‐based MOF had been proven to be a robust motif that was amenable to carboxylate ligand exchange.^45^ In order to further confirm that the amine group play a key role in enhancing the photocatalytic ability, we partly substituted BDC in MIL‐88B (Fe) by BDC–NH_2_ to obtain iron(III)‐based MOFs with mixed BDC and BDC–NH_2_ ligands. (See Experimental Section.) Three mixed iron(III)‐based MOFs were synthesized by varying the ratio of BDC and BDC–NH_2_ linkers, which were named as MOF‐1, MOF‐2, and MOF‐3, i.e., 15%, 30%, and 60% molar ratio of BDC in MIL‐88B (Fe) structure is replaced with BDC–NH_2_, respectively. As shown in Figure S10, Supporting Information, the three prepared mixed iron(III)‐based MOFs exhibited the similar XRD patterns, and were in good agreement with the simulated MIL‐88B structure. SEM images (Figure S11, Supporting Information) showed that the mixed iron(III)‐based MOFs remained the similar morphologies compared with MIL‐88B (Fe) and NH_2_–MIL‐88B (Fe). **Figure** **5**a displays the diffuse‐reflectance UV/vis spectrum of the prepared MIL‐88B (Fe), NH_2_–MIL‐88B (Fe), and mixed MIL‐88B (Fe). It shows that the introduction of amine group in the organic linker of iron(III)‐based MOF can enhance its light absorption in the visible region. This observation indicates that the more amine group incorporated into the iron(III)‐based MOF, the more electron–hole pairs can be generated via excitation of amine functionality under visible‐light irradiation, which might lead to enhance photocatalytic activity. Figure 5b shows the transient photocurrent responses of MIL‐88B (Fe), NH_2_–MIL‐88B (Fe), and mixed MIL‐88B (Fe) under intermittent visible light illumination. It can be seen that the photocurrent density of iron(III)‐based MOF with 100%, 60%, 30%, 15%, and 0% BDC–NH_2_ incorporation are about 6, 5, 3.5, 3, and 2 μA cm^−2^, respectively. The incorporation of amine group in the iron(III)‐based MOF can enhance the photocurrent significantly. This indicates that the separation efficiency of photoinduced electron–hole (e^−^–h^+^) pairs and the lifetime of the photogenerated charge carriers are improved, and this can be explained by the excitation of amine‐functionalized organic linker and then the excited electrons transfer to Fe_3_‐μ_3_‐oxo clusters.

**Scheme 1:**
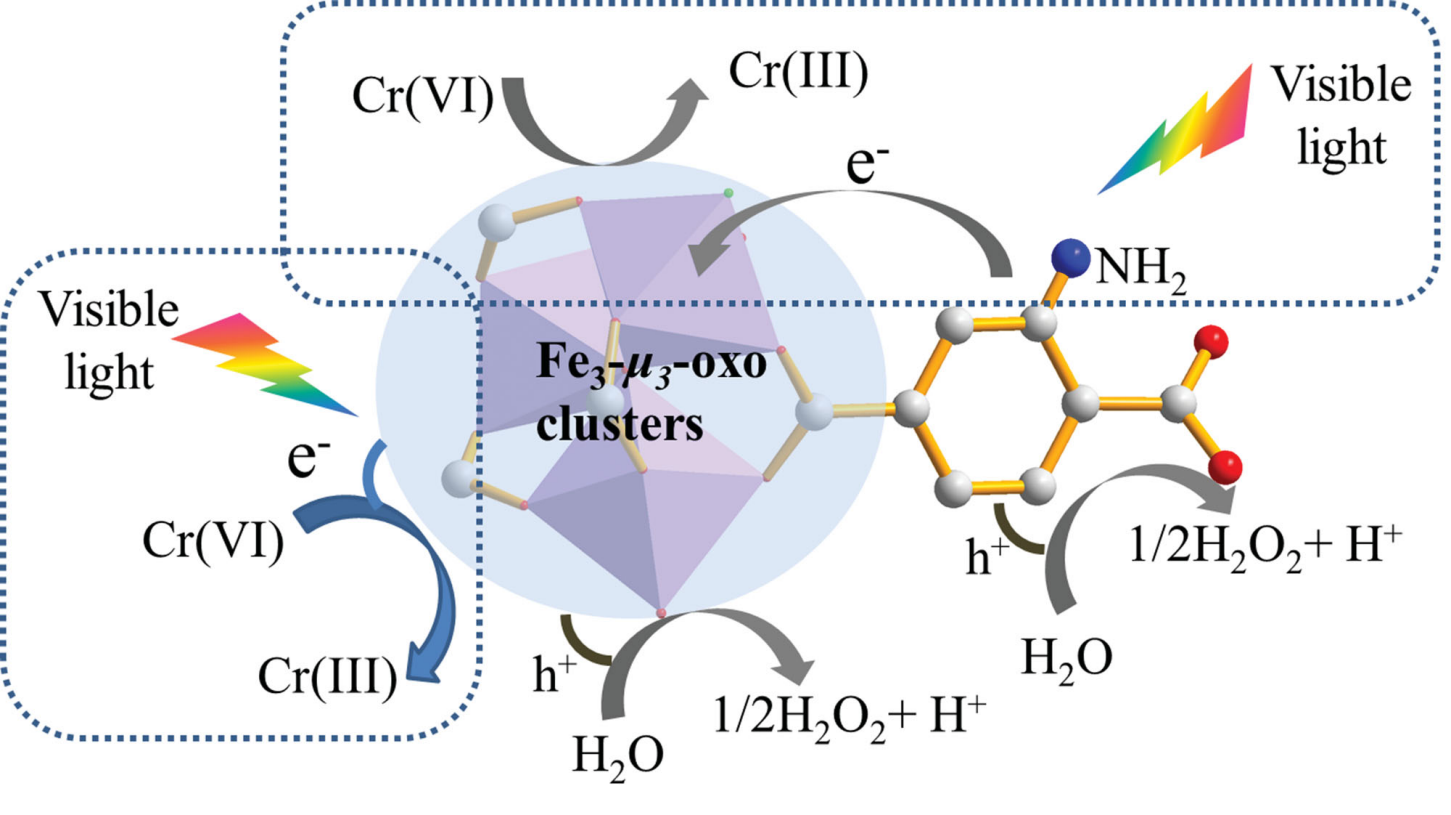
Proposed dual excitation pathways mechanism for photocatalytic reduction of Cr(VI) over NH_2_–MIL‐88B (Fe).

**Figure 6:**
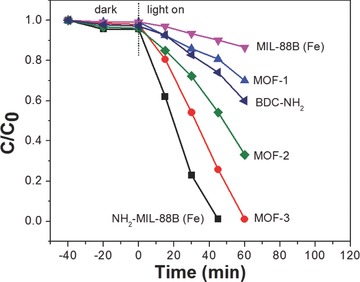
Reduction profiles of photocatalytic reduction of Cr(VI) over MIL‐88B (Fe), NH_2_–MIL‐88B (Fe), mixed MIL‐88B (Fe), and organic linker BDC–NH_2_. Reaction condition: 20 mg photocatalyst, 40 mL of 8 ppm Cr(VI), reaction temperature is 30 °C, pH = 2.

The influence of amine group incorporated in the iron(III)‐based MOF on the visible light photocatalytic activity was investigated by testing the photocatalytic performance for Cr(VI) reduction over MIL‐88B (Fe), NH_2_–MIL‐88B (Fe), and mixed MIL‐88B (Fe). Meanwhile, the photocatalytic ability of organic linker BDC–NH_2_ was also performed. As expected, the MIL‐88B (Fe) photocatalyst with higher amine groups incorporation exhibited better photocatalytic performance for Cr(VI) reduction under visible light irradiation (**Figure** **6**), which is due to the enhanced light absorption and efficient electron transfer as discussed above. Interestingly, organic linker BDC–NH_2_ also exhibits photocatalytic activity for Cr(VI) reduction, which demonstrates that the amine functionality can be excited under visible‐light irradiation. Significantly, NH_2_–MIL‐88B (Fe) shows much higher activity than that of BDC–NH_2_ and MIL‐88B (Fe), which means that the electron transfer phenomenon plays a key role in enhancing the photocatalytic activity, because the electron transfer can reduce the chance for electron–hole pairs recombination. On the basis of the above discussion, a mechanism of the photocatalytic reduction of Cr(VI) over NH_2_–MIL‐88B (Fe) was proposed, as shown in **Scheme** **1**. Under visible‐light irradiation, both of amine‐functionalized organic linker and Fe_3_‐μ_3_‐oxo clusters in the NH_2_–MIL‐88B (Fe) structure were excited. The electrons from the excited Fe_3_‐μ_3_‐oxo clusters can reduce the Cr(VI) to Cr(III), and at the same time, the photogenerated electrons in the organic linker transfer to the Fe_3_‐μ_3_‐oxo clusters, which is also responsible for the reduction of Cr(VI). The introduction of amine group in the MIL‐88B (Fe) structure can promote the electron transfer and reduce the chance for electron–hole pairs recombination, thus result in the enhanced photocatalytic performance.

Since the amine functionality in NH_2_–MIL‐88B (Fe) have a great effect on the photocatalytic performance for the ­reduction of Cr(VI), it is very interesting to investigate the photocatalytic performance of other iron(III)‐based MOFs photocatalysts. MIL‐53 (Fe) and MIL‐101 (Fe) were selected because these two kinds of iron(III)‐based MOFs have been proven to be stable photocatalysts and both contain the BDC organic linker similar to that in MIL‐88B (Fe) but have different structures.^34^ Moreover, amine‐functionalized NH_2_–MIL‐53 (Fe) and NH_2_–MIL‐101 (Fe) can also be obtained through organic linker substitution. The formation of these MOFs have been confirmed by their XRD patterns (Figure S12, Supporting Information). Those XRD patterns were in good agreement with as‐synthesized iron(III)‐based MOFs that were previously reported.^46^, ^47^, ^48^ The photocatalytic reduction of Cr(VI) was also investigated over MIL‐53 (Fe), NH_2_–MIL‐53 (Fe), MIL‐101 (Fe), and NH_2_–MIL‐101 (Fe). Amine‐functionalized NH_2_–MIL‐53 (Fe) shows an enhanced visible light absorption and improved photocatalytic performance compared to MIL‐53 (Fe) without amine functionalization (Figures S13a and S14, Supporting Information). The same trend was also observed for MIL‐101 (Fe) MOF with and without amine functionalization (Figures S13b and S14, Supporting Information). This observation indicated that amine functionalization in iron(III)‐based MOFs could be a general method to improve the photocatalytic performance for the reduction of Cr(VI). However, we observed a significant difference in the activity enhancement for NH_2_–MIL‐53 (Fe), NH_2_–MIL‐101 (Fe), and NH_2_–MIL‐88B (Fe) compared with their unfunctionalized MOFs. The difference may be ascribed to the different ­structures and electron transfer efficiency of these iron(III)‐based MOFs photocatalysts.

## 3 Conclusion

In summary, iron(III)‐based MOFs structured as MIL‐88B structure were successfully produced through a rapid microwave‐assisted solvothermal synthesis method. The prepared amine‐functionalized NH_2_–MIL‐88B (Fe) shows high stability and efficiency for the photocatalytic Cr(VI) reduction under visible‐light irradiation. The visible‐light activity of NH_2_–MIL‐88B (Fe) was even higher than that of P25 under UV‐visible light irradiation. It was observed that the incorporation of amine group in the iron(III)‐based MOFs resulted in enhanced photocatalytic activity for Cr(VI) reduction, which was due to the enhanced light absorption and efficient charge transfer from organic linker to Fe_3_‐μ_3_‐oxo clusters. The effect of amine functionalization in the other two iron(III)‐based MOFs (MIL‐53 (Fe) and MIL‐101 (Fe)) on the visible light photocatalytic activity for the reduction of Cr(VI) was also studied. The same trend was observed, namely, all of the amine‐functionalized MOFs showed enhanced activity for photocatalytic Cr(VI) reduction compared with those of their parent MOFs. This work elucidated the mechanism of iron(III)‐based MOFs as photocatalysts for Cr(VI) reduction. It also proved that MOFs can serve as stable and efficient photocatalysts and the light harvesting efficiency can be flexibly tuned by simply modifying the organic linker in the MOFs structure.

## 4 Experimental Section

*Materials*: *N*,*N*‐dimethylformamide (DMF), ferric chloride hexahydrate (FeCl_3_·6H_2_O), BDC, and BDC–NH_2_ were purchased from Wako Co. All reagents were analytical grade and used without further purification. Deionized water (18.2 MΩ) used throughout all experiments was produced using a Millipore Direct‐Q System.

*Synthesis of Photocatalysts*: Iron(III)‐based MOFs MIL‐88B (Fe) and amine‐functionalized MIL‐88B (Fe) (NH_2_–MIL‐88B (Fe)) crystals were produced from a rapid microwave‐assisted solvothermal synthesis method. For the synthesis of MIL‐88B (Fe), BDC (3 mmol, 0.49839 g) and FeCl_3_·6H_2_O (3 mmol, 0.8109 g) were dissolved in DMF (50 mL). After vigorously stirred for 10 min, this solution was placed in a sealed vessel and heated by microwave at 400 W for 15 min at 150 °C. After cooling to room temperature, the resulting particles were isolated by centrifugation, washed with ethanol and deionized water several times, and then dried at 343 K under vacuum overnight. NH_2_–MIL‐88B (Fe) was achieved by replacing BDC with BDC–NH_2_, while keeping all other synthetic conditions unchanged. Mixed MIL‐88B (Fe) was prepared using a similar procedure as above except that a certain percentage of BDC was replaced with BDC–NH_2_. Other two visible light active MOF photocatalysts NH_2_–Uio‐66–Zr and NH_2_–MIL‐125–Ti were synthesized according to the literatures previously reported.^32^, ^33^ Iron(III)‐based MOFs MIL‐53, MIL‐101, and their amine‐functionalized derivatives were synthesized according to the literatures.^46^, ^47^, ^48^ Briefly, MIL‐53 (Fe) was synthesized from a mixture of FeCl_3_·6H_2_O, BDC, and DMF with a molar ratio of 1:1:280, then the mixture was heated at 150 °C for 15 h with a heating ramp of 1 h. The amine‐functionalized NH_2_–MIL‐53 (Fe) was synthesized by hydrothermal treatment of a mixture of 0.9 g (5 mmol) of BDC–NH_2_, 50 mL of H_2_O, and 1.35 g (5 mmol) of FeCl_3_·6H_2_O at 150 °C for three days. MIL‐101 (Fe) was synthesized via a hydrothermal reaction of a mixture of FeCl_3_·6H_2_O, BDC, and DMF with a molar ratio of 2:1:560 at 110 °C for 24 h. NH_2_–MIL‐101(Fe) was prepared similar to MIL‐101(Fe) except that BDC was replaced by BDC–NH_2_. All the as‐synthesized iron(III)‐based MOFs were obtained by centrifugation, washed with water and ethanol several times, and then dried at 343 K under vacuum overnight. g‐C_3_N_4_ was prepared by heating melamine to 550 °C for 4 h under air condition in a crucible.

*Characterization*: The XRD patterns of the prepared samples were recorded on an X‐ray diffractometer (Shimadzu, XRD‐7000) with monochromatized Cu Ka radiation (*λ* = 1.54178 Å), under 40 kV and 30 mA. UV–visible diffuse reflectance spectra were measured on UV–visible spectrophotometer (Hitachi U‐3900) with BaSO_4_ as the reflectance standard reference. XPS were performed on Thermo ESCALAB250 using monochromatized Al Kα at hυ = 1486.6 eV. The morphology and size of the samples were observed with a SEM (JSM‐6701F, JEOL Co., Japan). The PL spectra were recorded on a JASCO FP‐6500 spectrofluorometer. ESR characterizations were carried out with JEOL JES‐FA‐200 at room temperature under vacuum.

*Photoelectrochemical Measurement*: To prepare the photoelectrodes, 10 mg iron(III)‐based MOFs was added into 1 mL of ethanol. The as‐prepared solution was stirred for 1 h to ensure that the iron(III)‐based MOFs were uniformly dispersed in the solution. 10 μL of the 10 mg mL^−1^ iron(III)‐based MOFs solution was dropped onto the surface of ITO substrate, which had an exposed area of 1.0 × 1.0 cm^2^, and then dried under vacuum condition for 1 h at 80 °C. This step was repeated five times to ensure a uniform coverage of iron(III)‐based MOFs on ITO. Photocurrent measurements were performed with an electrochemical station (ALS/CH model 650A, Japan) using a three‐electrode mode with 0.5 m Na_2_SO_4_ solution (pH = 7.0) as the electrolyte. The iron(III)‐based MOFs photoanodes were used as working electrodes; a Pt wire served as a counter electrode. The Ag/AgCl electrode was used as the reference electrodes. A 500 W Xe lamp with a UV‐cutoff filter (420 nm) was used as the visible light source. The Mott–Schottky measurements were performed at a frequency of 1000 Hz in the dark.

*Photocatalytic Experiments*: The photocatalytic activities of iron(III)‐based MOFs were evaluated by photocatalytic reduction of Cr(VI) under visible‐light irradiation of a 300 W Xe arc lamp with a 420 nm cutoff filter and cooling water filter. Potassium dichromate (K_2_Cr_2_O_7_) was selected as a Cr(VI) compound. The photocatalytic reduction of Cr(VI) was performed at about 30 °C in a quartz reactor containing 20 mg photocatalyst and 40 mL of Cr(VI) solution (8 mg L^−1^ based on Cr in a dilute K_2_Cr_2_O_7_ solution). The solution was stirred for 40 min in the dark to reach adsorption equilibrium and then was exposed to visible light irradiation. The Cr content in the reaction solution was determined using the DPC method.^17^ The photocatalytic efficiency was determined by dividing *C*/*C*_0_, where *C* is the remained Cr(VI) concentration and *C*_0_ is the starting Cr(VI) concentration.

## Supporting information

As a service to our authors and readers, this journal provides supporting information supplied by the authors. Such materials are peer reviewed and may be re‐organized for online delivery, but are not copy‐edited or typeset. Technical support issues arising from supporting information (other than missing files) should be addressed to the authors.

Supplementary
