# Supplementary material for: An Amine‐Functionalized Iron(III) Metal–Organic Framework as Efficient Visible‐Light Photocatalyst for Cr(VI) Reduction
Source: Adv Sci (Weinh). 2015 Feb 9;2(3):1500006. doi: 10.1002/advs.201500006 (PMC5115284; doi:10.1002/advs.201500006)
Supplement: Supplementary file 1 — Supplementary [file ADVS-2-0k-s001.pdf]

Copyright WILEY-VCH Verlag GmbH & Co. KGaA, 69469 Weinheim, Germany, 2015.

ADVANCED SCIENCE
Open Access

Supporting Information

for *Adv. Sci.*, DOI: 10.1002/advs. 201500006

An Amine-Functionalized Iron(III) Metal–Organic Framework as Efficient Visible-Light Photocatalyst for Cr(VI) Reduction

*Li Shi, Tao Wang, Huabin Zhang, Kun Chang, Xianguang Meng, Huimin Liu, and Jinhua Ye**

Supporting Information

An Amine-functionalized Iron(III) Metal-organic Framework as Efficient Visible-light Photocatalyst for Cr(VI) Reduction

Li Shi[a,b,c], *Tao Wang*[b,c], *Huabin Zhang*[b,c], *Kun Chang* [b,c] *Xianguang Meng*[a,b,c], *Huimin Liu*[b,c], *Jinhua Ye*[a,b,c,d,*]

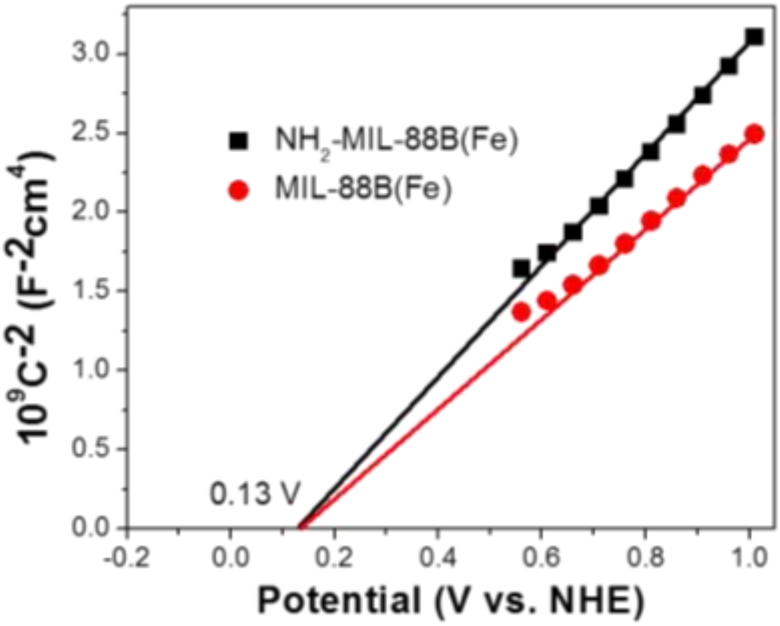

Figure S1. Mott-Schottky plot of MIL-88B (Fe) and NH_2-MIL-88B(Fe).

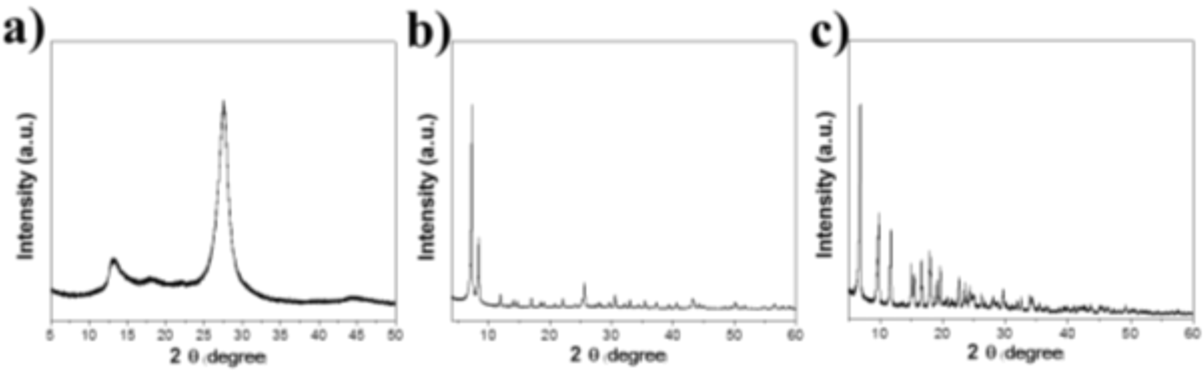

Figure S2. XRD patterns of (a) g-C_3N_4, (B) NH_2-Uio-66-Zr and (C) NH_2-MIL-125-Ti.

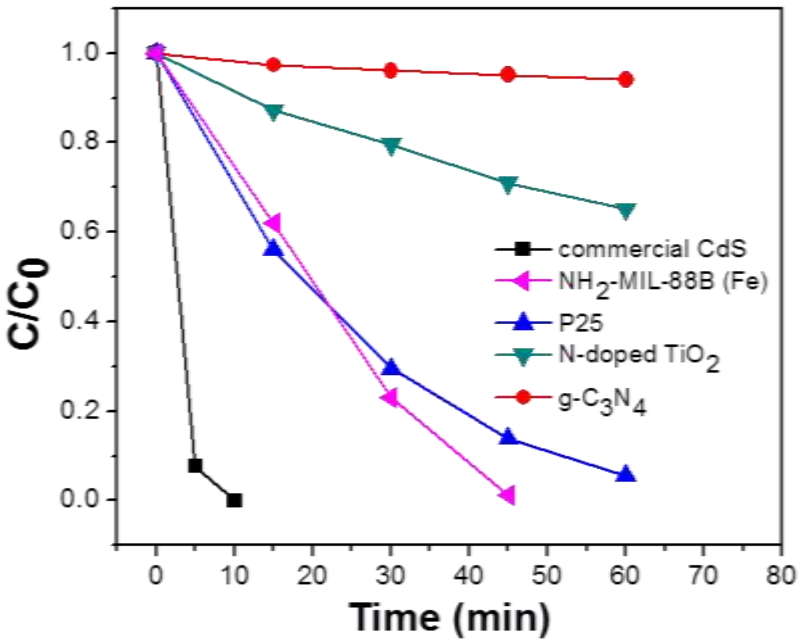

Figure S3. Reduction profiles of photocatalytic reduction of Cr(VI) over various photocatalysts. Reaction condition: 20 mg photocatalyst, 40 ml of 8 ppm Cr(VI), reaction temperature is 30°C, pH=2. P25 is conducted in UV-visible light and the other photocatalysts are conducted in visible light. The solution is stirred for 40 min in the dark and then is exposed to visible light irradiation.

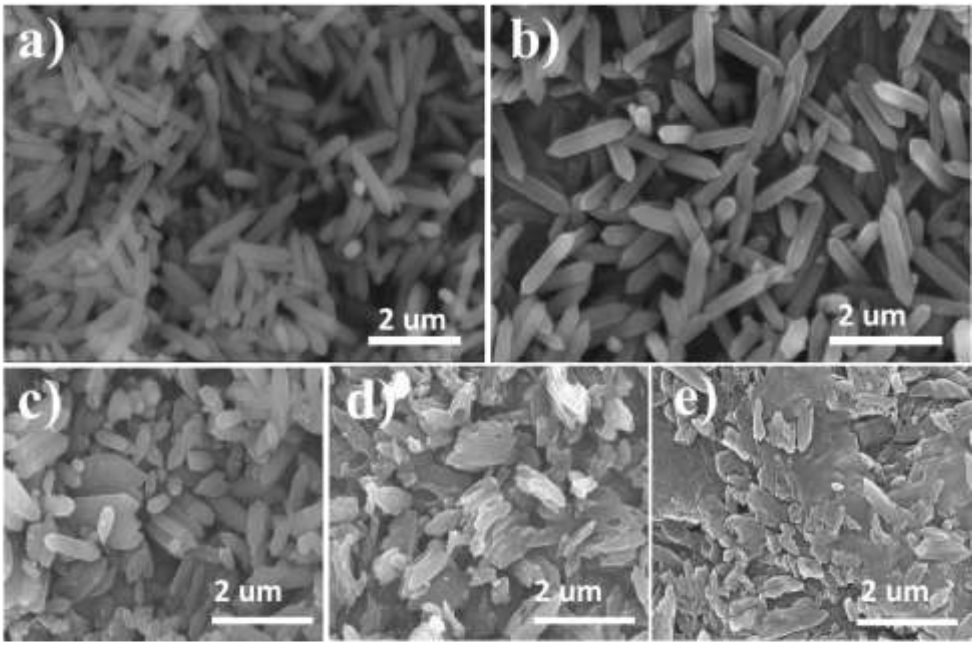

Figure S4. SEM images of NH2-MIL-88B (Fe) after photocatalytic reaction at different pH values: (a) 4; (b) 3; (c) 2; (d) 1.7; (e) 1.5.

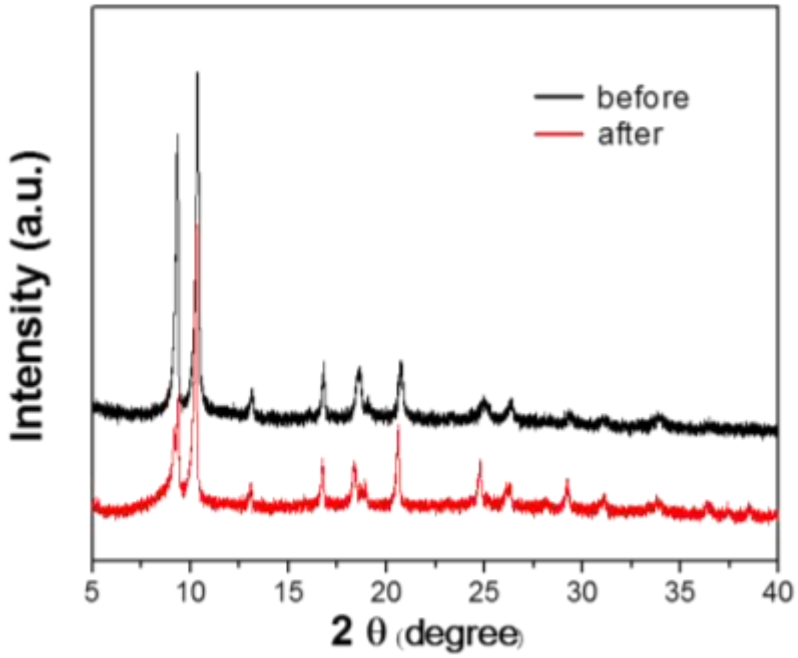

Figure S5. XRD patterns of NH_2-MIL-88B (Fe) before and after photocatalytic reaction.

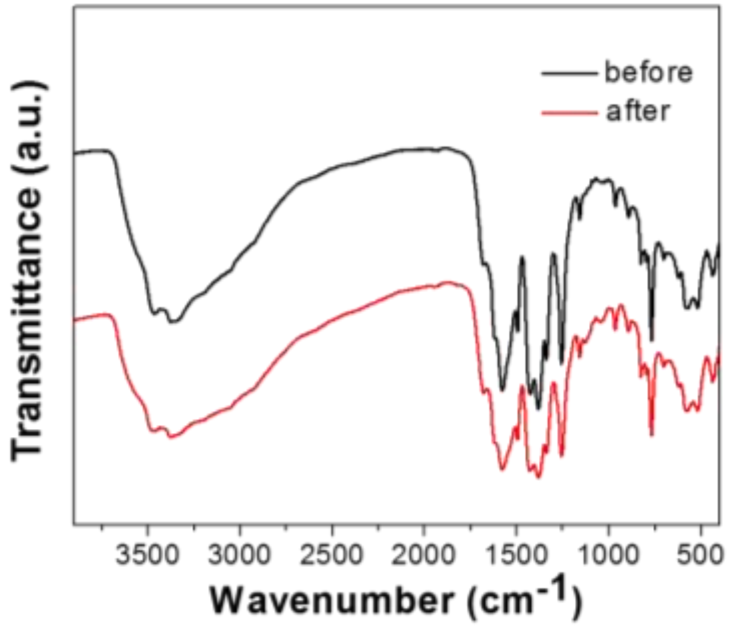

Figure S6. FTIR spectra of NH_2-MIL-88B (Fe) before and after photocatalytic reaction.

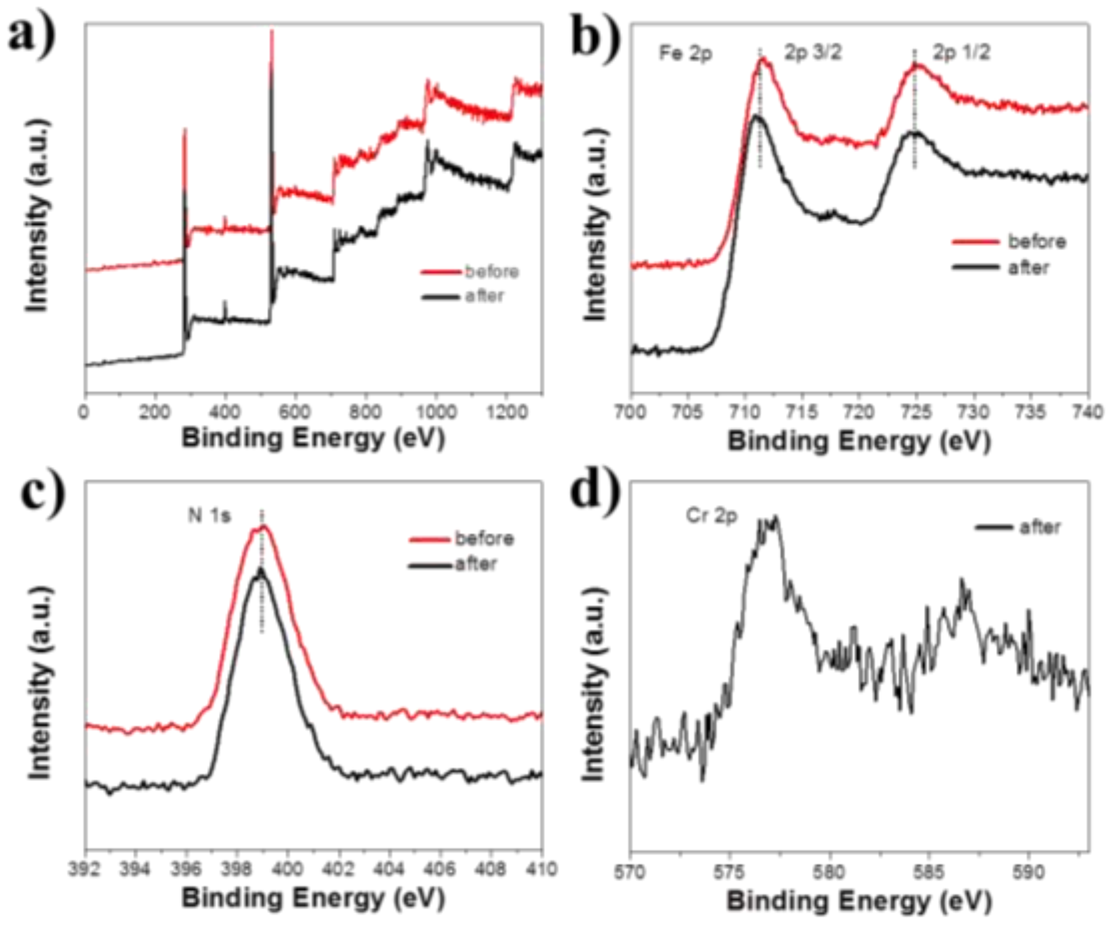

Figure S7. XPS patterns of NH_2-MIL-88B (Fe) before and after photocatalytic reaction.

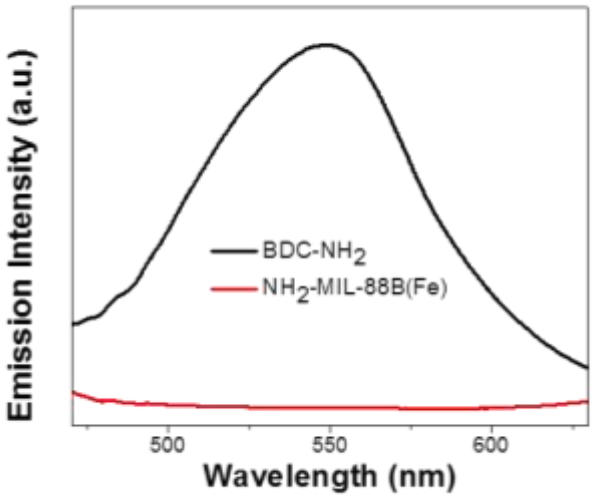

Figure S8. PL properties of NH_2-MIL-88B (Fe) and BDC-NH_2 at excitation wavelength of 350 nm. The PL study was carried out on NH_2-MIL-88B (Fe) and BDC-NH_2 powders.

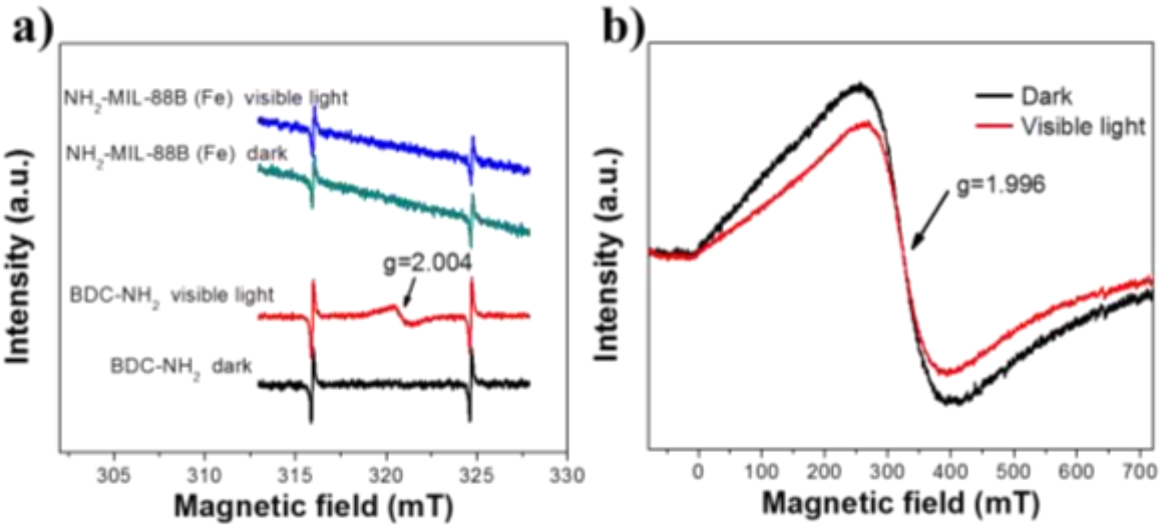

Figure S9. (a) ESR spectra of BDC-NH_2 and NH_2-MIL-88B (Fe) measured before and after visible light irradiation; (b) ESR spectra of NH_2-MIL-88B (Fe) measured before and after visible light irradiation.

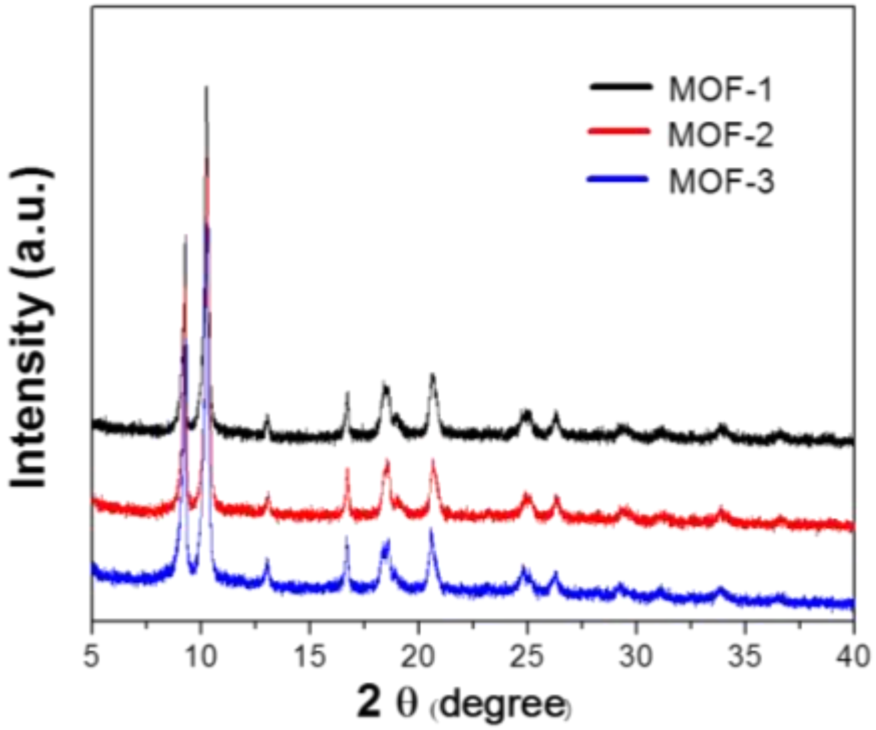

Figure S10. XRD patterns of mixed MIL-88B (Fe) with different percentage of BDC-NH_2 incorporation.

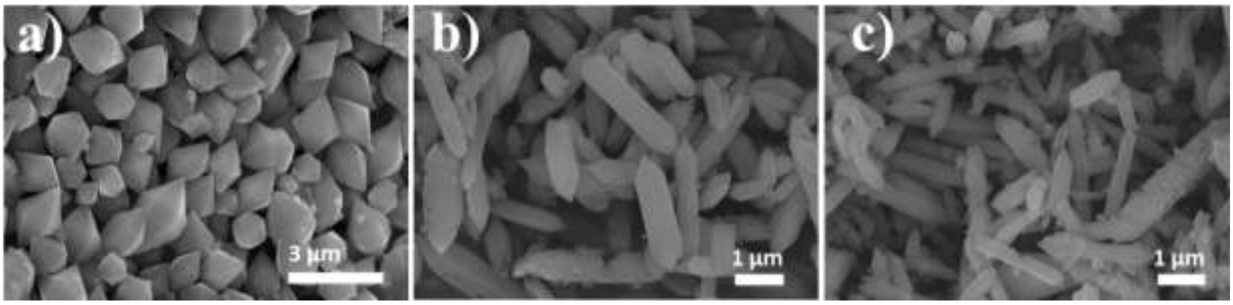

Figure S11. SEM images of (a) MOF-1; (b) MOF-2 and (c) MOF-3.

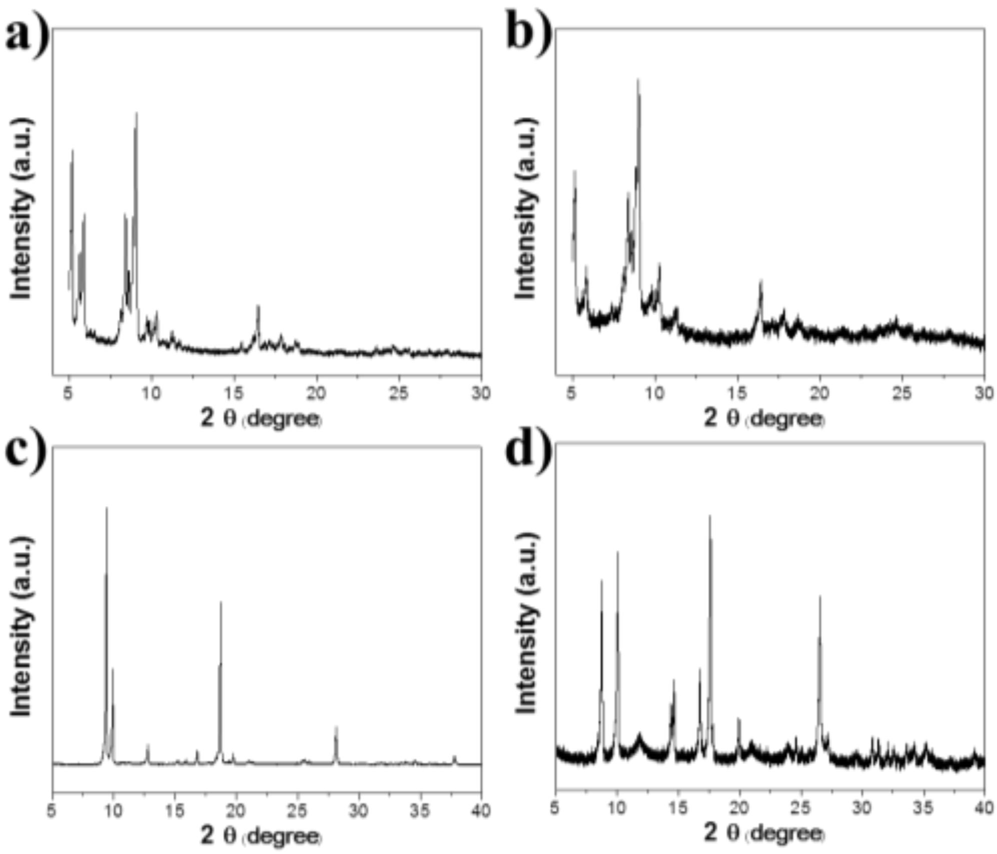

Figure S12. XRD patterns of as-synthesized (a) MIL-101 (Fe); (b) NH_2-MIL-101 (Fe); (c) MIL-53 (Fe) and (d) NH_2-MIL-53 (Fe).

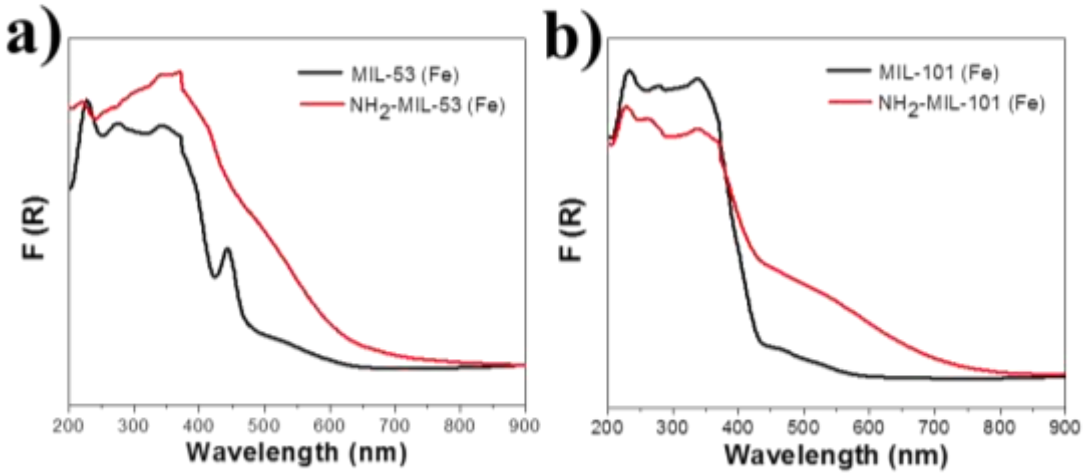

Figure S13. The diffuse-reflectance UV/Vis spectrums of (a) MIL-53 (Fe) and (b) MIL-101 (Fe) with and without amine functionalization.

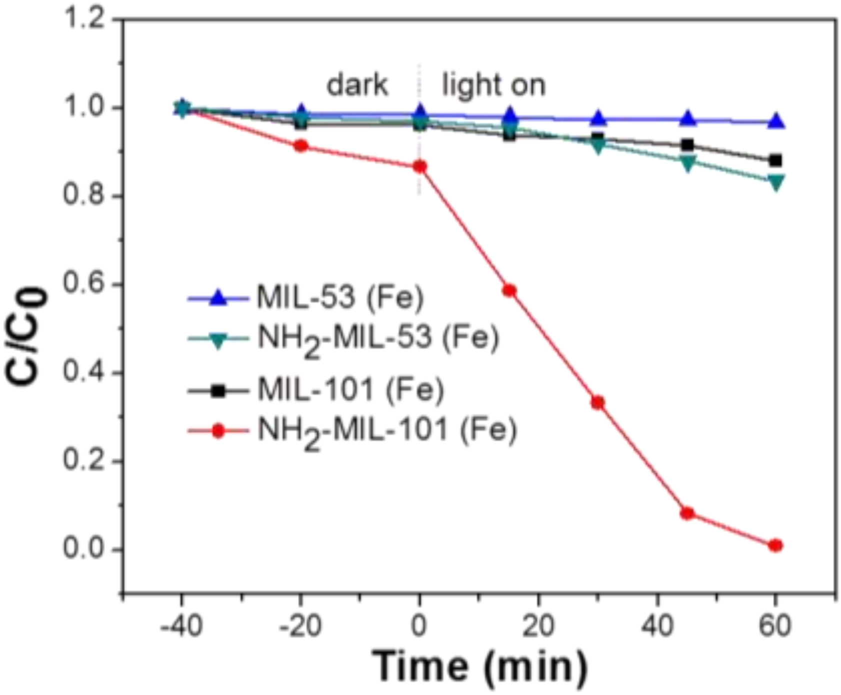

Figure S14. Reduction profiles of photocatalytic reduction of Cr(VI) over MIL-101 (Fe), NH_2-MIL-101 (Fe), MIL-53 (Fe) and NH_2-MIL-53 (Fe). Reaction condition: 20 mg photocatalyst, 40 ml of 8 ppm Cr(VI), reaction temperature is 30℃, pH=2.
